# Regulation mechanism and bioactivity characteristic of surfactin homologues with C14 and C15 fatty acid chains

**DOI:** 10.1186/s12934-024-02373-6

**Published:** 2024-03-27

**Authors:** Yumeng Su, Ling Gao, Chenyu Li, Liang Wang, Huimin Zhou, Chenhao Zhang, Xiaole Xia

**Affiliations:** Key Laboratory of Industrial Biotechnology, Ministry of Education, School of Biotechnology, Jiangnan University, 1800 Lihu Road, Wuxi, Jiangsu 214122 People’s Republic of China

**Keywords:** Surfactin, Homologues, Regulation mechanism, Transcriptome analysis, Bioactivity

## Abstract

**Background:**

Surfactin, a green lipopeptide bio-surfactant, exhibits excellent surface, hemolytic, antibacterial, and emulsifying activities. However, a lack of clear understanding of the synthesis regulation mechanism of surfactin homologue components has hindered the customized production of surfactin products with different biological activities.

**Results:**

In this study, exogenous valine and 2-methylbutyric acid supplementation significantly facilitated the production of C14–C15 surfactin proportions (up to 75% or more), with a positive correlation between the homologue proportion and fortified concentration. Subsequently, the branched-chain amino acid degradation pathway and the glutamate synthesis pathway are identified as critical pathways in regulating C14–C15 surfactin synthesis by transcriptome analysis. Overexpression of genes *bkdAB* and *glnA* resulted in a 1.4-fold and 1.3-fold increase in C14 surfactin, respectively. Finally, the C14-rich surfactin was observed to significantly enhance emulsification activity, achieving an EI_24_ exceeding 60% against hexadecane, while simultaneously reducing hemolytic activity. Conversely, the C15-rich surfactin demonstrated an increase in both hemolytic and antibacterial activities.

**Conclusion:**

This study presents the first evidence of a potential connection between surfactin homologue synthesis and the conversion of glutamate and glutamine, providing a theoretical basis for targeting the synthesis regulation and structure–activity relationships of surfactin and other lipopeptide compounds.

**Graphical Abstract:**

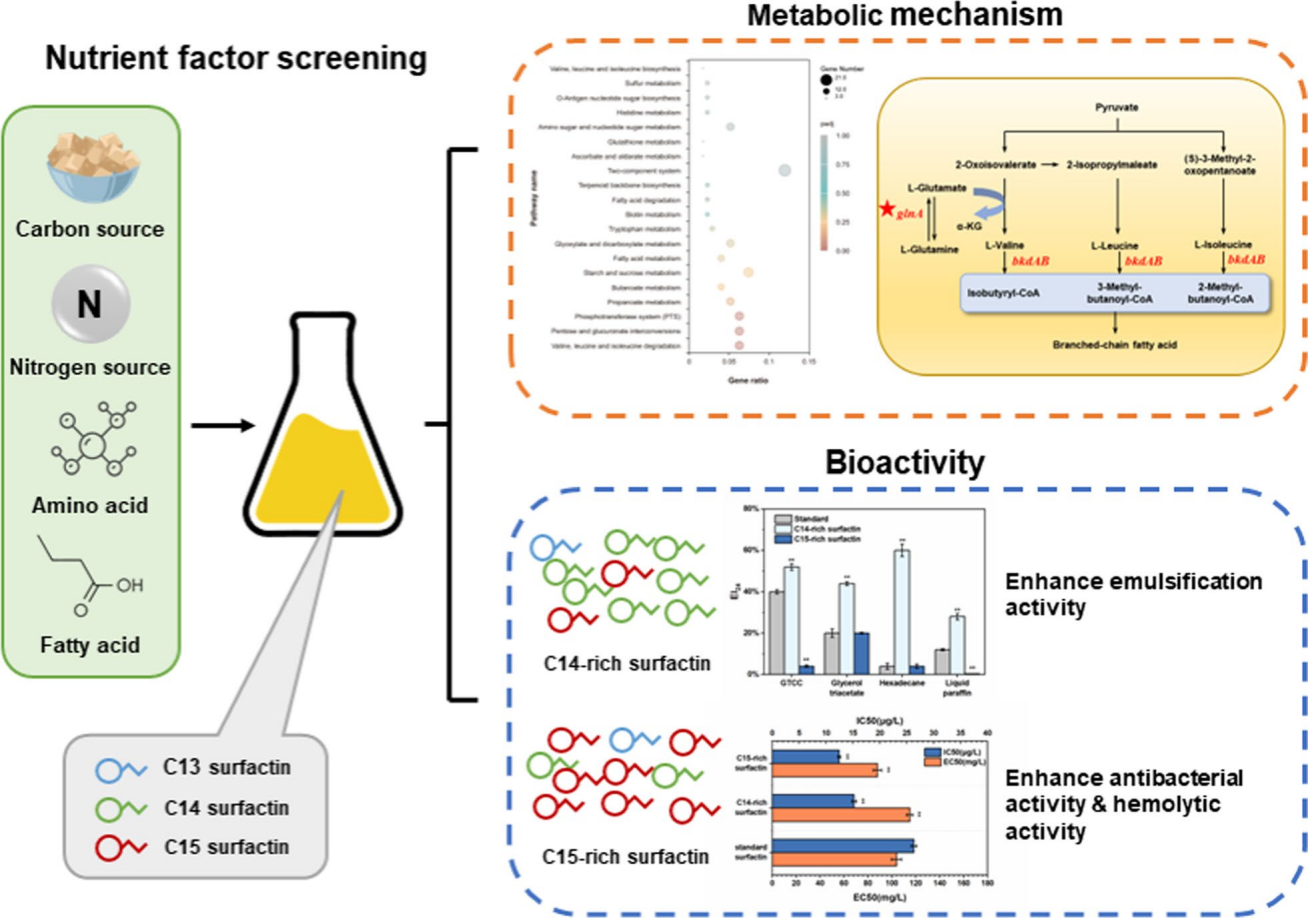

**Supplementary Information:**

The online version contains supplementary material available at 10.1186/s12934-024-02373-6.

## Background

Surfactin, a green and biodegradable surfactant, is one of the most effective biobased surfactants, possessing extensive application value in biology, medicine, daily chemicals, and food fields [1]. Surfactin is a secondary metabolite of *B. subtilis*, consisting of 7 amino acids and a β-hydroxy fatty acid with a side chain length of 13–15 carbon atoms [2]. Previous research suggested a strong association between the functional capability of lipopeptide and the fatty acid chain length connected to the peptide [3]. C14 surfactin has been verified to possess a better foaming capability and quality than C13–C15 surfactin [4], whereas the antibacterial and hemolytic properties increase proportionally as the fatty acid chain lengthens [5]. Therefore, different surfactin homologues are well-suited for diverse industrial applications based on their distinct biological activities. Surfactin with a high C14 composition is optimal for use as an emulsifier and solubilizer in food and daily chemicals [6]. For applications such as drug delivery and oil extraction, a surfactin with a high C15 composition offers greater advantages [7, 8].

However, most of the surfactin produced by bacteria is a mixture of various homologues. Uneven distribution of homologue proportion and low purity limit industrial use of surfactin. Currently, the methods to modify the homologue proportion of surfactin are almost based on branched-chain amino acids. Different amino acids were added into the *B. subtilis TD7* culture medium, with arginine, glutamine, and valine significantly increasing the proportion of surfactin variants with even-numbered β-hydroxy fatty acids [9]. Using gene knockout techniques, the percentage of straight-chain C14 surfactin isomers was increased up to 53% by modifying the branched-chain fatty acid pathway in *B. subtilis *[10]. In addition, the amount of straight-chain C14 surfactin was promoted to 55%–60% by overexpressing the plant acyl carrier protein (ACP) thioesterase (*BTE*) [11]. However, the above strategies have merely achieved a 60% uptick in the proportion of C14 surfactin components, which is insufficient to meet industrial production demands. There is no investigation into the influence of potentially crucial genes in the global metabolic network on the directed-oriented synthesis of distinct surfactin homologues. Transcriptomic technologies have opened new avenues for guiding the construction of high-production strains by providing a deeper understanding of the genetic background of microorganisms, and allow rapid selection of effective metabolic engineering targets. Bacitracin yield was significantly increased by rational engineering of *B. licheniformis* on the basis of transcriptome data, demonstrating that *TEII*_*bac*_ (encoding type II thioesterase) and oxygen supply are critical for bacitracin production [12].

This study provides novel insights into the mechanism of surfactin synthesis to achieve effectively targeted synthesis of C14–C15 surfactin. To identify the crucial nutritional factors affecting surfactin homologue synthesis, the nutrients in fermentation medium were systematically evaluated using different carbon sources, inorganic nitrogen sources, amino acids, and fatty acids. Subsequently, the cellular metabolism mechanisms associated with the targeted biosynthesis of C14–C15 surfactin in *B. subtilis Q1* were further elucidated through global transcriptome analysis. Finally, the structures and bioactivities of purified C14-rich surfactin and C15-rich surfactin were analyzed, demonstrating a significant enhancement in the emulsifying capacity and antimicrobial efficiency of surfactin.

## Methods

### Plasmids and strain construction

Table 1 lists the strains and plasmids used in this study. The original strain producing surfactin was *B. subtilis Q1* and plasmid construction experiments were performed using *E. coli JM109* as the host. *B. subtilis Q1* is a high surfactin-producing strain obtained in our laboratory in the early stages, derived from the model bacterium *B. subtilis 168*. Genetic modifications were made by knocking out the *pps* operon (encoding the lipopeptide plipastatin synthetase) and replacing the native PsrfA promoter regulating the surfactin synthetase gene cluster, leading to increased surfactin production. The high yield of surfactin ensures prominent peak profiles during HPLC analysis and facilitates the isolation and purification of C14-rich and C15-rich surfactin for bioactivity determination.

**Table 1:** Strains and plasmids used in this research

Strain or plasmid	Description	Source
Strains		
*E. coli JM109*	The cloning host	This lab
*B. subtilis Q1*	Starting strain, *B. subtilis 168* derivative, surfactin high-yielding strain	This lab
*B. subtilis Q1-2*	*B. subtilis Q1* derivative, ΔbkdAB	This lab
Plasmids		
pHT01-bkdAA	AmpR(*E. coli*), ChlR(*B. subtilis*), P43-bkdAA	This study
pHT01-bkdAB	AmpR(*E. coli*), ChlR(*B. subtilis*), P43-bkdAB	This study
pHT01-phoD	AmpR(*E. coli*), ChlR(*B. subtilis*), P43-phoD	This study
pHT01-phoB	AmpR(*E. coli*), ChlR(*B. subtilis*), P43-phoB	This study
pHT01-glnA	AmpR(*E. coli*), ChlR(*B. subtilis*), P43-glnA	This study
pHT01-glnJ	AmpR(*E. coli*), ChlR(*B. subtilis*), P43-glnJ	This study
pKS2-bkdAB	KanR(*E. coli*), KanR(*B. subtilis*)	This study

The genes *bkdAA*, *bkdAB*, *phoD*, *phoB*, *glnA* and *glnJ* from *B. subtilis 168* were amplified, purified, and ligated into pHT01 with promoter P43 (with KpnI and BamHI as the restriction sites) resulting into the recombinant plasmids pHT01-P43-bkdAA, pHT01-P43-bkdAB, pHT01-P43-phoD, pHT01-P43-phoB, pHT01-P43-glnA and pHT01-P43-glnJ, respectively. The upstream (*bkdAB-L*) and downstream (*bkdAB-R*) DNA sequences of the gene *bkdAB* from *B. subtilis Q1* were amplify, purified, and ligated into pKS2 (with KpnI and salI as the restriction sites), resulting into the recombinant plasmid pKS2-bkdAB. PrimeSTAR^®^ Max DNA Polymerase and restriction enzymes purchased from TAKARA (Dalian, China). The kits used for gene purification, plasmid extraction and One-step cloning were purchased from Vazyme (Nanjing, China). All DNA constructs were sequenced by Sangon Biotech (Shanghai, China).

The *E. coli JM109* was cultured in 50 mL LB medium (10 g/L sodium chloride, 5 g/L yeast extract, 10 g/L peptone) at 37 °C and 200 rev·min^−1^. The seed culture of *B. subtilis Q1* was LB medium containing a relevant antibiotic at 37 °C and 200 rev·min^−1^. Next, 7% culture solution was transferred to 30 mL modified fermentation medium (70 g/L brown sugar, 3 g/L yeast extract, 17 g/L NaNO_3_, 0.15 g/L MgSO_4_·7H_2_O, 0.006 g/L FeSO_4_·7H_2_O, 0.006 g/L MnSO_4_·H_2_O, 23.4 g/L Na_2_HPO_4_, 3.4 g/L citric acid) incubated in a shaker at 37 ℃ and 200 rev·min^−1^. IPTG was added at 1 mM for 1 h of incubation in a shaker, followed by continuous fermentation up to 24 h. Before inoculation, ampicillin, chloromycetin and/or kanamycin were added for the selection of recombinant strains at final concentrations of 100, 5 and 50 μg/mL, respectively.

### Isolation, purification, and analysis of surfactin

The acid precipitation method was used to extract and purify surfactin. Following fermentation, the cells were separated from the media by centrifugation at 5000 rpm for 10 min. Then, add 1:1 ethanol to the supernatant, mix and shake well, adjust the pH to 6.0 –7.0, and leave for 1 h to remove the protein. Separation of ethanol by spin distillation at 40 hPa, 30 °C.

To achieve a pH of 2.0 for acid precipitation, 6 mol/L HCl was added to the supernatant. The fermentation broth obtained after rotary evaporation, add 6 mol/L HCl to adjust the pH to 2 for acid precipitation. Centrifugation was used to collect the acid precipitate for 10 min at 5000 g. After using 5 mol/L NaOH to adjust the final pH down to 7.0, the mixture was vacuum-dried in a freeze drier.

The surfactin concentration was determined using high-performance liquid chromatography (HPLC) on a chromaster instrument (HITACHI, Japan) equipped with an ZORBAX Eclipse Plus C18 column (4.6 × 250 mm, 5 μm) and a DA detector (HITACHI, Japan). The mobile phase was composed of acetonitrile and water, with 1‰ trifluoroacetic acid at a flow rate of 0.8 mL/min. Standard surfactin (99%) was purchased from Shanghai Yuanye Bio-Technology Co., Ltd. The cell growth was monitored by measuring the optical density at 600 nm (OD_600_).

Surfactin components were further analyzed by UPLC–MS (WATERS ACQUITY) coupled with a single quadrupole MS (WATERS MALDI SYNAPT Q-TOF) on a BEH C18 column (2.1 × 50 mm 1.7 µm) using a method based on the acetonitrile/water (acidified with 0.1% formic acid) gradient. Ionization and source conditions were set as follows: source temperature, 100 °C; desolvation temperature, 400 °C; Cone Gas Flow, 50 L/h; voltage, 1800 V.

### Transcriptome analysis and RT-qPCR

Control strains were cultured in fermentation medium without additional nutrients, and experimental strains were cultured in fermentation medium exogenously supplemented with 4 g/L valine and 2 g/L 2-methylbutyric acid, respectively. Additionally, 30 mL of fermentation broth was used to construct samples C14–C15. The cultured organisms were then centrifuged and collected at 10,000 rpm at 4 °C, repeat the procedure after resuspension with sterile PBS, frozen in liquid nitrogen, and sent to Novogene (Beijing, China) for transcriptome analysis. And the samples are also sent to GENEWIZ (Suzhou, China) for RT-qPCR analysis.

DESeq R was used to do a differential expression analysis of two conditions. DESeq offers statistical procedures based on a negative binomial distribution model for identifying differential expression in digital gene expression data. The Benjamini and Hochberg method for regulating the false discovery rate was applied to the generated P-values. Genes identified as differentially expressed by DESeq were those with an adjusted P-value < 0.005.

### Measurement of bioactivity

#### Measurement of hemolytic activity

The purified C14 surfactin, C15 surfactin, and standard surfactin were prepared into different concentrations of the to-be-tested substances by successive twofold gradient dilutions of PBS solution. 100 μL of the test solution was taken in a 2 mL centrifuge tube, and 100 μL of 4% (v/v) sheep erythrocyte suspension was added and mixed well. The negative control was PBS solution, and the positive control was 0.1 (v/v) Trilatone-PBS solution. The well mixed reaction system was incubated at 37 °C for 1 h. After centrifugation at 5000 rpm for 5 min, 100 μL of supernatant was taken in a 96-well plate, and its absorbance at 540 nm was measured. Two technical parallels were set for each condition.

#### Measurement of antibacterial activity

Using *Micrococcus luteus* as the indicator bacteria, the activation was carried out by three-zone delineation, and the single colony on the plate was picked and incubated in LB liquid medium at 37 ℃, 200 rpm for 12 h, then diluted to the bacterial solution with OD_600_ of 0.1 by LB. The purified C14-rich surfactin, C15-rich surfactin, and standard surfactin were prepared into different concentrations of the test material by successive twofold gradient dilution in LB medium. 100 μL of the test solution and 100 μL of *Micrococcus luteus* were taken in sterile 96-well plates respectively. The absorbance values at 600 nm (OD_600_) were measured after 24 h of static incubation in a 37 ℃ incubator. Water was used as a negative control, and two technical parallels were set for each condition. The inhibitory activity was calculated as antibacterial activity = [As-An]/ An*100%. As, and a respectively represent the absorbance of the test solution and the negative control at 600 nm.

#### Measurement of emulsifying activity

The purified C14-rich surfactin, C15-rich surfactin, and standard surfactin were prepared as aqueous solutions with a concentration of 50 mg/L. 1.5 mL of each test sample solution was added to glass tubes with calibrations, followed by the addition of 1 mL of neodecanoic triglyceride, triethyl glyceride, hexadecane, and liquid paraffin, respectively. The mixtures were thoroughly shaken and allowed to stand for 24 h to ensure proper emulsification. The emulsification index (EI_24_) was then calculated ${\text{EI}}_{{{24}}} \, = \frac{Height\,of\,emulsion\,layer}{{Total\,height\,of\,liquid\,layer}}\, \times \,100\%$.

## Results

### Influence of externally supplemented amino acids and fatty acids on the proportion of surfactin homologues

**Fig. 1:**
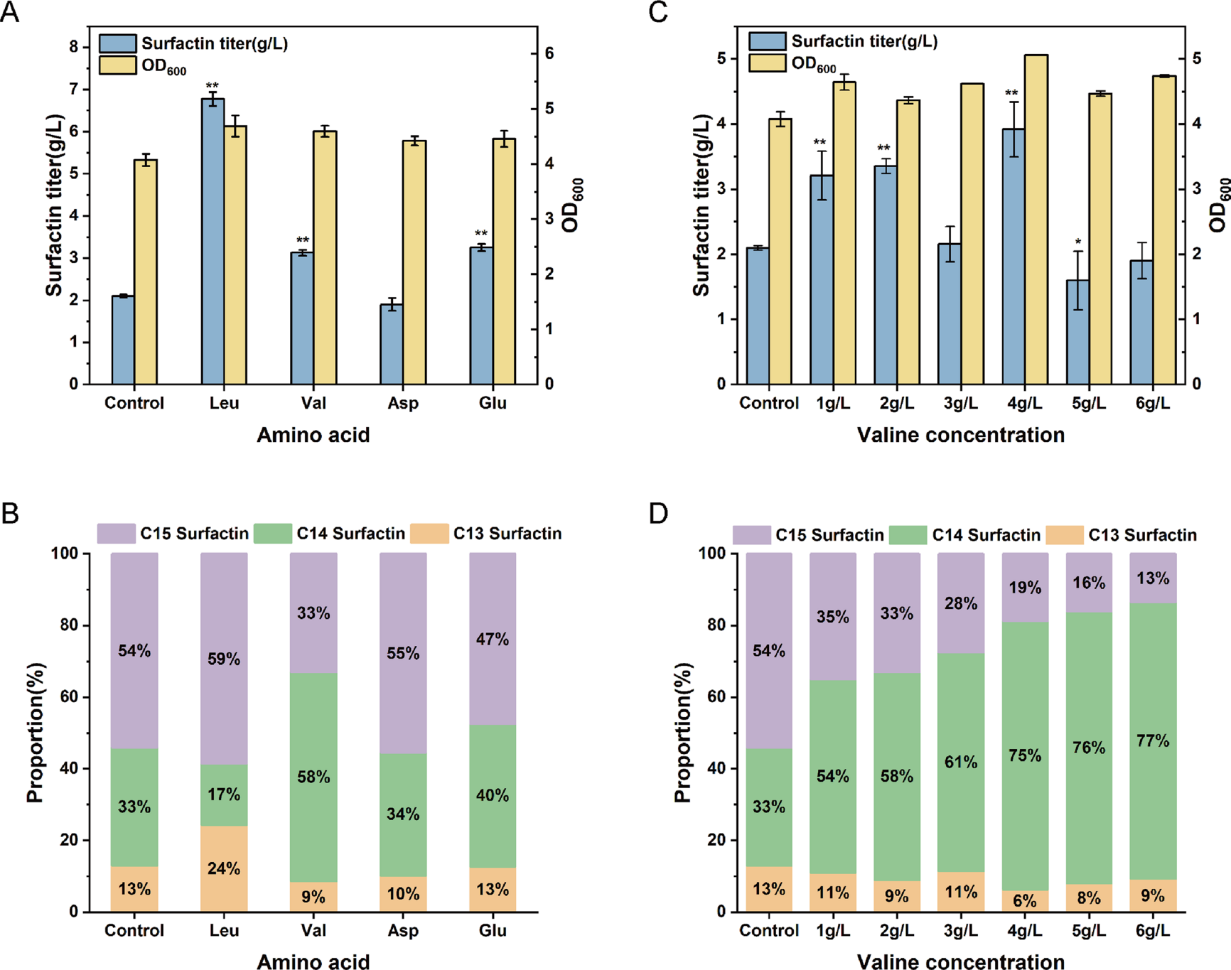
Enhancement of C14 surfactin proportion by exogenous addition of valine in *B. subtilis Q1*. **A** Surfactin titer and OD_600_ of *B. subtilis Q1* at different amino acid addition. **B** Surfactin homologues proportion of *B. subtilis Q1* at different amino acid addition. **C** Surfactin titer and OD_600_ of *B. subtilis Q1* at different valine concentration. **D** Surfactin homologues proportion of *B. subtilis Q1* at different Valine concentration

In our previous work, the effects of different carbon sources, nitrogen sources, and buffer systems on surfactin and homologues were investigated (Additional file 1: Fig. S1, 2). Using sucrose as the carbon source, NaNO_3_ as the inorganic nitrogen source, and a Na_2_HPO_4_-citric acid buffer system at pH 7.0, we obtained a yield of surfactin of 2.1 g/L. The corresponding proportions of surfactin homologues were 13% (C13 surfactin), 33% (C14 surfactin), and 54% (C15 surfactin), respectively. The result is consistent with the proportion of surfactin standards purchased from Shanghai Yuanye Bio-Technology Co., Ltd. The activity of surfactin is influenced by its various homologues. To investigate the nutritional factors that affect the composition of surfactin homologues, this study supplied various precursor amino and fatty acids during the initial fermentation process. The fermentation results showed that 2 g/L concentration of amino acid supplementation, such as leucine, valine, and glutamate had a positive impact on surfactin production (Fig. 1A). Especially, leucine supplementation at 2 g/L generated a 3.2-fold increase in surfactin yield, up to 6.8 g/L compared with the control group without amino acid addition. Interestingly, the supplementation of valine and glutamate both displayed a positive effect on the proportion of C14 surfactin homologues (Fig. 1B). Moreover, the proportion of C14 surfactin homologues was enhanced with the increase in valine concentration (Fig. 1D). The highest proportion of C14 surfactin homologues could reach up to 77% in the fermentation condition supplemented with 6 g/L valine. However, a high concentration of valine (≥ 5 g/L) significantly reduced cell growth and surfactin production (Fig. 1C). Elevated valine concentrations augment the metabolic burden and promote the synthesis of other amino acids, ultimately resulting in energy and resource deficits that adversely affect bacterial growth and surfactin production [13, 14]. The optimal 4 g/L valine supplementation could generate the highest surfactin production and 75% proportion of C14 surfactin homologues. These results indicated that adjusting the concentration of valine not only improved surfactin production, but also fine-tuned the proportion of C14 surfactin homologues.

**Fig. 2:**
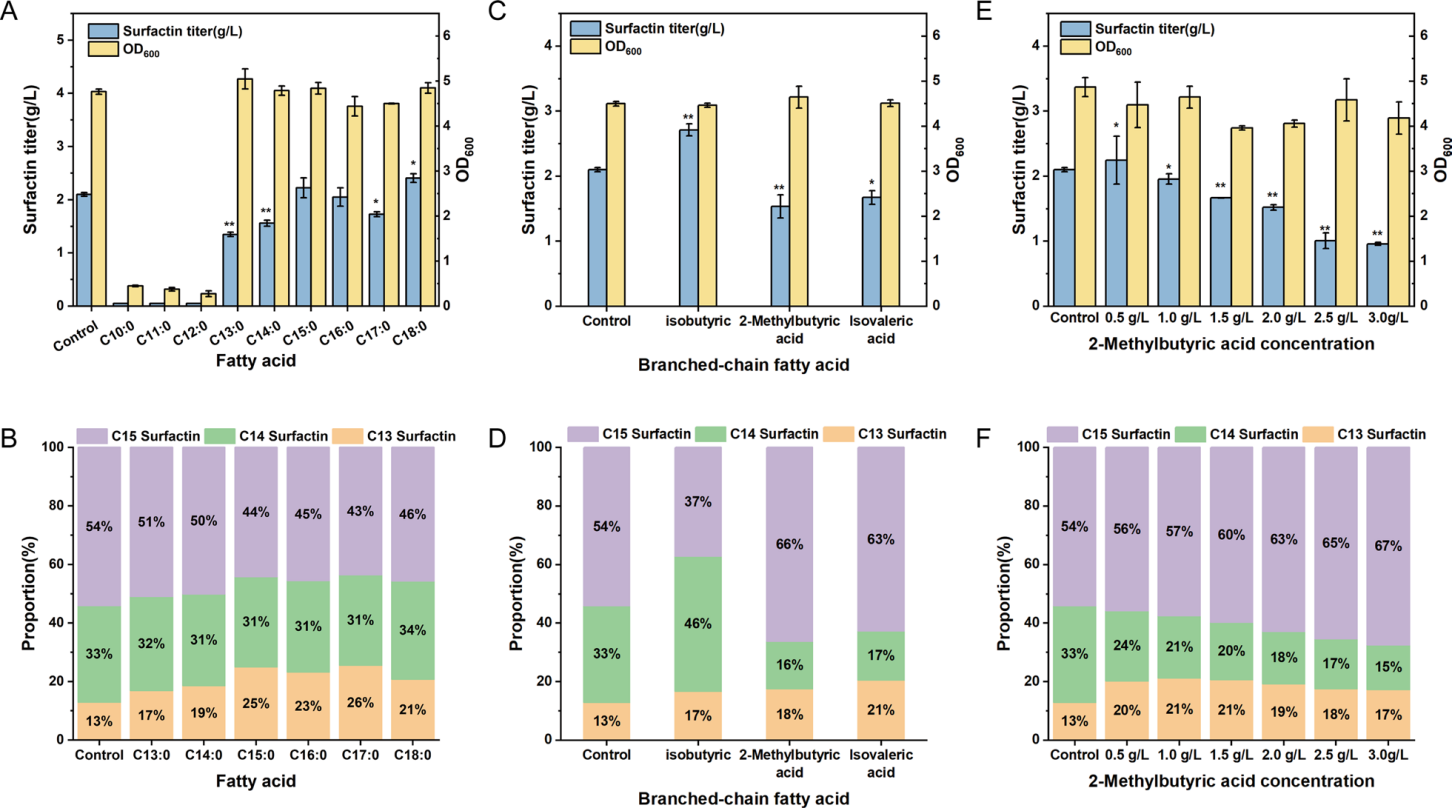
Enhancement of C15 surfactin proportion by exogenous addition of 2-methylbutyric acid in *B. subtilis Q1*. **A** Surfactin titer and OD_600_ of *B. subtilis Q1* at straight-chain fatty acid (10C–18C) addition. **B** Surfactin homologues proportion of *B. subtilis Q1* at straight-chain fatty acid (10C–18C) addition. **C** Surfactin titer and OD600 of *B. subtilis Q1* at different branched-chain fatty acid addition. **D** Surfactin homologues proportion of *B. subtilis Q1* different branched-chain fatty acid addition. **E** Surfactin titer and OD_600_ of *B. subtilis Q1* at different 2-methylbutyric acid concentration. **F** Surfactin homologues proportion of *B. subtilis Q1* at different 2-methylbutyric acid concentration

Serving as essential precursors for surfactin synthesis, fatty acids were presumably the most direct way of regulating lipid acyl chain lengths [15]. However, there was no significant effect on the composition of the surfactin homologues through adding straight-chain fatty acids (C10–C18) (Fig. 2B). And the addition of straight-chain fatty acids with carbon lengths of 10, 11, and 12 exerted a toxic effect on cell growth, hindering bacterial proliferation (Fig. 2A). Notably, the addition of branched short-chain fatty acids such as isobutyric acid, isovaleric acid, and 2-methylbutyric acid had an obvious effect on synthesis of surfactin homologues (Fig. 2D). The isobutyric acid showed an improved surfactin production and an increase of C14 surfactin homologues, while the 2-methylbutyric acid and isovaleric acid displayed a decreased surfactin production and an increase of C15 surfactin homologues. Taking an example of added 2-methylbutyric acid, the proportion of C15 surfactin homologues was enhanced with the increase in branched short-chain fatty acid concentration, reaching a maximum of approximately 70%. Therefore, enhancing the proportion of C14 surfactin could be achieved by controlling exogenous valine and glutamate, as well as even-chain branched fatty acids (such as isobutyric acid). Similarly, enhancing the proportion of C15 surfactin could be obtained by regulating odd-chain branched-chain fatty acids (such as 2-methylbutyric acid and isovaleric acid).

### Regulation mechanism of surfactin homologues biosynthesis through comparative transcriptomic analysis

Valine and 2-methylbutyric acid were selected for further mechanistic study due to their significant effects on surfactin homologue ratios, with valine enhancing the proportion of C14 surfactin and 2-methylbutyric acid elevating the proportion of C15 surfactin. To explore the metabolic mechanisms of biosynthesis between C14-rich surfactin and C15-rich surfactin, global transcriptional changes in different shake-flask fermentation conditions were analyzed. *B. subtilis Q1* grown in basal fermentation media was used as a control. The strains grown in fermentation medium with a supplement of 4 g/L valine and 2 g/L 2-methylbutyric acid were numbered sample C14–C15, respectively. To identify the genes responsible for the high yield of C14 surfactin and C15 surfactin, differentially expressed genes in two samples with statistical differences (padj < 0.005) in comparison with the control group (|log_2_FoldChange|> = 1.0) were screened.

**Fig. 3:**
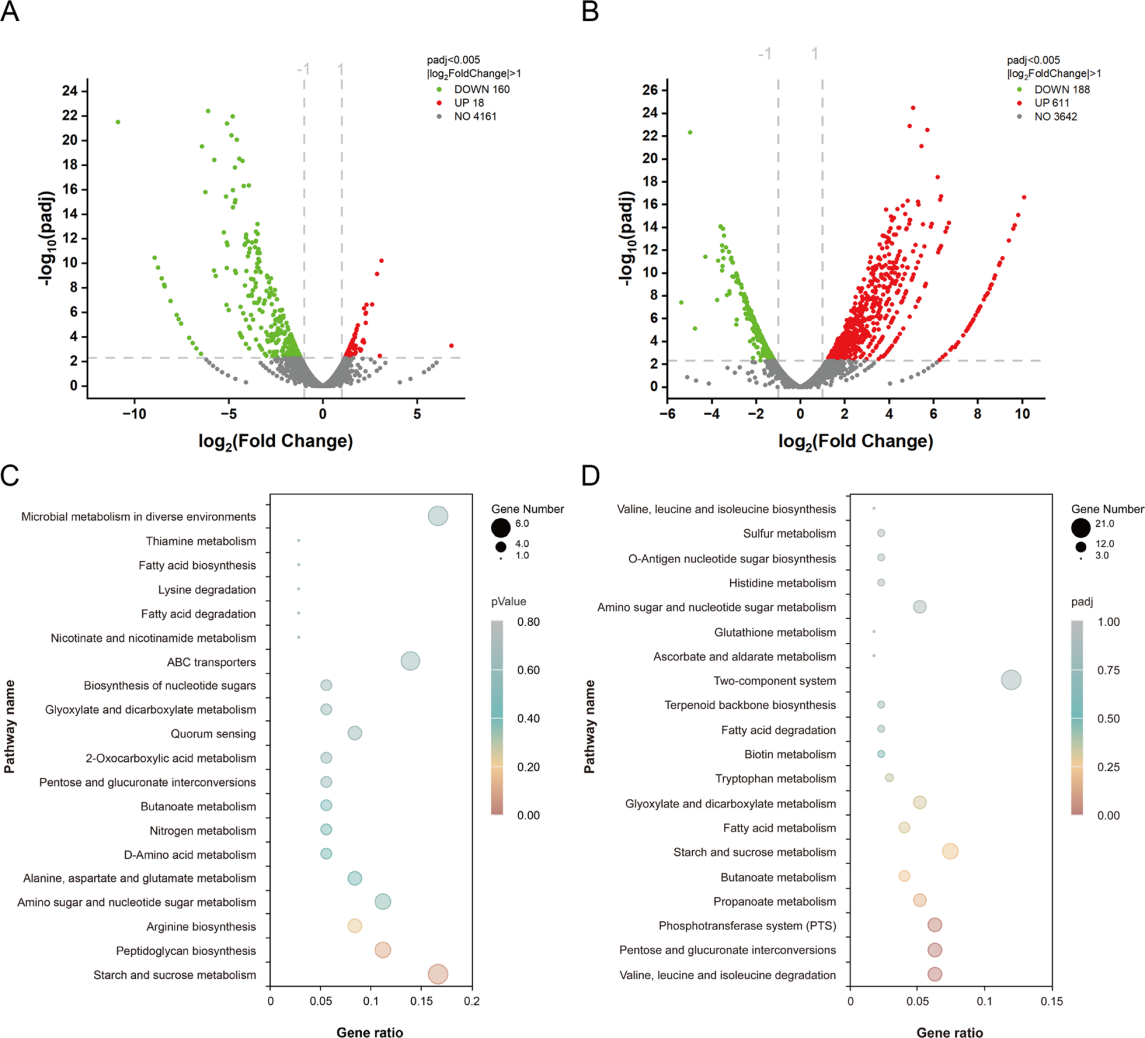
Transcriptomic analysis of sample C14 and C15. **A** Volcano plot of sample C14. **B** Volcano plot of sample C15. **C** KEGG enrichment factor bubble chart of sample C14. **D** KEGG enrichment factor bubble chart of sample C15

A total of 18 genes were upregulated and 178 genes were downregulated in sample C14 (Fig. 3A), and 611 genes were upregulated and 188 genes were downregulated in sample C15 (Fig. 3B). The genes with significant expression differences were analyzed using KEGG pathway enrichment. These genes in sample C14 were mainly involved in the starch and sucrose metabolism, ABC transporter, and arginine biosynthesis, and in sample C15 were mainly involved in the two-component system, phosphotransferase system (PTS), and the degradation of branched-chain amino acids.

**Fig. 4:**
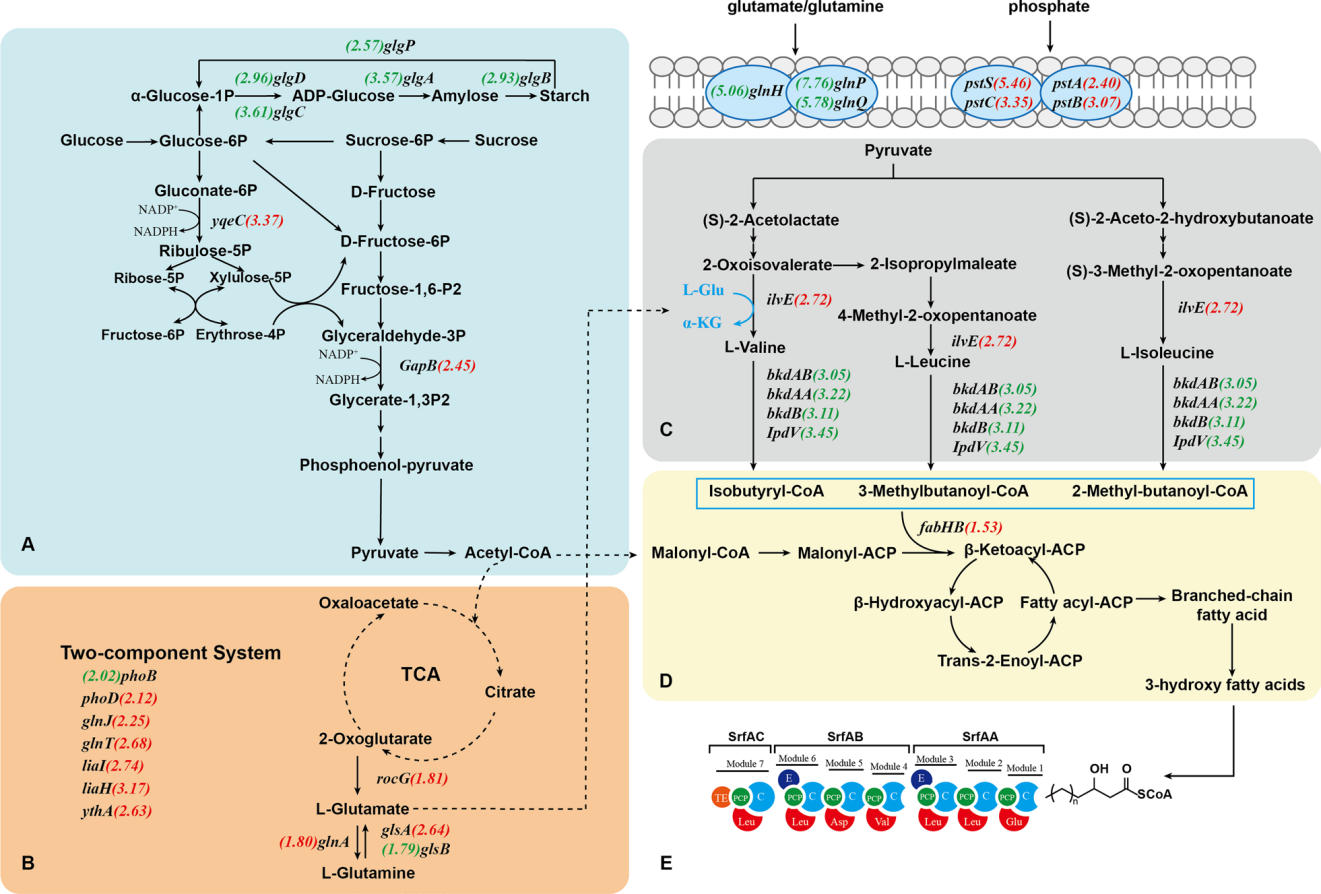
Surfactin synthesis pathway network reconstruction based on KEGG database. **A** Sucrose metabolism. **B** TCA cycle with Asp and Glu metabolism. **C** Branched-chain amino acid metabolism. **D** Fatty acid metabolism. **E** Surfactin biosynthesis. The numbers indicate the absolute value of log_2_FoldChange in transcript levels of relative genes in the sample C14 and C15 relative to those in control sample. The colors red and green show considerable upregulation and downregulation, respectively. The numbers at left and right side of the gene name indicate the transcriptome results of samples C14 vs control and C15 vs control, respectively

To further analyze changes in the global metabolism of samples C14–C15, the altered expression levels of genes in the major metabolic pathways of surfactin synthesis were investigated. These pathways were reconstructed in modules, including sucrose metabolism, TCA cycle with Asp and Glu metabolism, branched-chain amino acid metabolism, and fatty acid metabolism (Fig. 4). In sample C14, genes involved in sucrose metabolism were generally downregulated, which not only affected sugar utilization but also had an impact on glycogen metabolism. Previous studies have supported the idea that bacterial glycogen storage and overaccumulation could lead to better growth or a higher OD_600_ value [16]. A study demonstrated that an *E. coli* mutant with a single gene disruption in *glgBXCAP*, a key gene cluster involved in glycogen metabolism, exhibited increased glycogen accumulation, which facilitated growth but reduced glucose consumption in liquid culture [17]. Similarly, in sample C14, the genes *glgA*, *glgC*, *glgD*, *glgB*, and *glgP* were all downregulated, resulting in glycogen accumulation. This finding suggested that the addition of valine may increase the OD_600_ by downregulating the genes according to glycogen enrichment.

The addition of excessive 2-methylbutyric acid can lead to an elevation of butyrate and butanol levels within cells through various metabolic pathways, which may have a negative effect on cell metabolism and survival. Previous research has been published showing that the butyrate stress response causes the expression of heat shock protein (HSP), which needs more ATP to provide energy to resist changes in the external environment [18]. In sample C15, the expression of 6-phosphogluconate dehydrogenase (encoded by *yqeC*) increased by a remarkable 10.3-fold, and glyceraldehyde-3-phosphate dehydrogenase (encoded by *gapB*) showed a 5.5-fold increase in sample C15. These two enzymes are crucial for the production of NADPH in the catalytic process of sucrose metabolism. Furthermore, the genes *ythA*, *liaI*, and *liaH*, identified as transcriptional regulators that enhance cell resistance through upregulation, were all found to be upregulated in sample C15 [19, 20]. These results suggested that the presence of 2-methylbutyric acid may impose growth restrictions on the strain, leading to a decrease in OD_600_ and necessitating additional energy consumption to sustain growth. This ultimately contributed to a reduction in surfactin production, as demonstrated by previous fermentation experiments.

Branched-chain acyl-CoA, such as 2-methylbutyric acid-CoA, 2-methylpropanoyl-CoA, and 3-methylbutanoyl-CoA, are derived from branched-chain amino acid degradation. In sample C15, genes involved in the branched-chain amino acid degradation pathway (*bkdAB*, *bkdAA*, *bkdB*, and *IpdV*) were significantly downregulated. This suggested that the addition of 2-methylbutyric acid resulted in feedback inhibition of precursor branched-chain amino acid degradation, leading to a decrease in the synthesis of even-chain branched-chain fatty acids derived from valine. In addition, the expression of β-ketoacyl-acyl carrier protein synthase (encoded by *fabHB*) increased by 2.9-fold. This enzyme is responsible for catalyzing the first reaction in the synthesis of the nascent carbon chain, which involves the condensation of malonyl-ACP with acyl-CoA to produce more odd-chain fatty acids [21]. C15 surfactin synthesis was enhanced and positively correlated with 2-methylbutyric acid addition, due to the higher odd-numbered fatty acid ratio in the pool. The findings of this study further demonstrated that adjusting the ratio of odd/even chains in the intracellular pool of branched-chain fatty acids can regulate the percentage of C15–C14 surfactin homologues.

First identified in *E. coli* and subsequently identified in numerous additional bacterial species, the *Pho* regulon is a global regulatory system involved in bacterial inorganic phosphate(Pi) control [22]. The *PhoPR* two-component regulators in control the expression of the *Pho* regulon genes, which are expressed in response to phosphate shortages [23]. These cellular reactions include raising the affinity of the phosphate transporter (encoded by *pstSCAB*) to enhance cellular phosphate intake and eliminating excess phosphate by secreting alkaline phosphatase (encoded by *phoA*, *phoB*, and *phoD*). The *PhoPR* two-component system has been reported to control the synthesis of secondary metabolites via phosphate [24] and regulate branched-chain amino acid biosynthesis to affect the synthesis of the lipopeptide fengycin [25]. Compared with the control sample, the *phoB* gene was down-regulated in sample C14, while the *phoD* gene and *pstSCAB* genes (including *pstC*, *pstA*, *pstS*, and *pstBB*) were all up-regulated in sample C15. Based on this result, there is speculation that *Pho* regulation may have an effect on the synthesis of different surfactin components.

In sample C14, glutamine synthetase (encoded by *glnA*) was upregulated, while glutaminase (encoded by *glsB*) was downregulated. The expression of glutaminase (encoded by *glsA*) and glutamate dehydrogenase (encoded by *rocG*) in sample C15 were both elevated. These changes in gene expression indicated that the conversion of glutamate to glutamine was enhanced in sample C14 but reversed in sample C15. Additionally, genes mediating glutamate import in sample C14, including *glnH*, *glnP*, and *glnQ*, were downregulated to limit glutamate input. In contrast, the glutamine utilization genes *glnJ* and *glnT*, annotated as components of the two-component system (TCS), were upregulated in sample C15 to consume glutamine and accumulate glutamate. Glutamate and glutamine are the primary amino group suppliers for all nitrogen-containing molecules, including other amino acids and building blocks for DNA and RNA synthesis [26]. In *B. subtilis*, glutamate serves as the amino group donor in most transamination reactions, including branched-chain amino acid [27]. According to the transcriptomics data and previous fermentation results with glutamate addition, it is hypothesized that the conversion of glutamate and glutamine mediated branched-chain amino acid metabolism, which in turn affects the formation of different surfactin homologues.

### Regulation of surfactin homologue synthesis by branched-chain amino acids and glutamate/glutamine metabolism in *B. subtilis Q1*

**Fig. 5:**
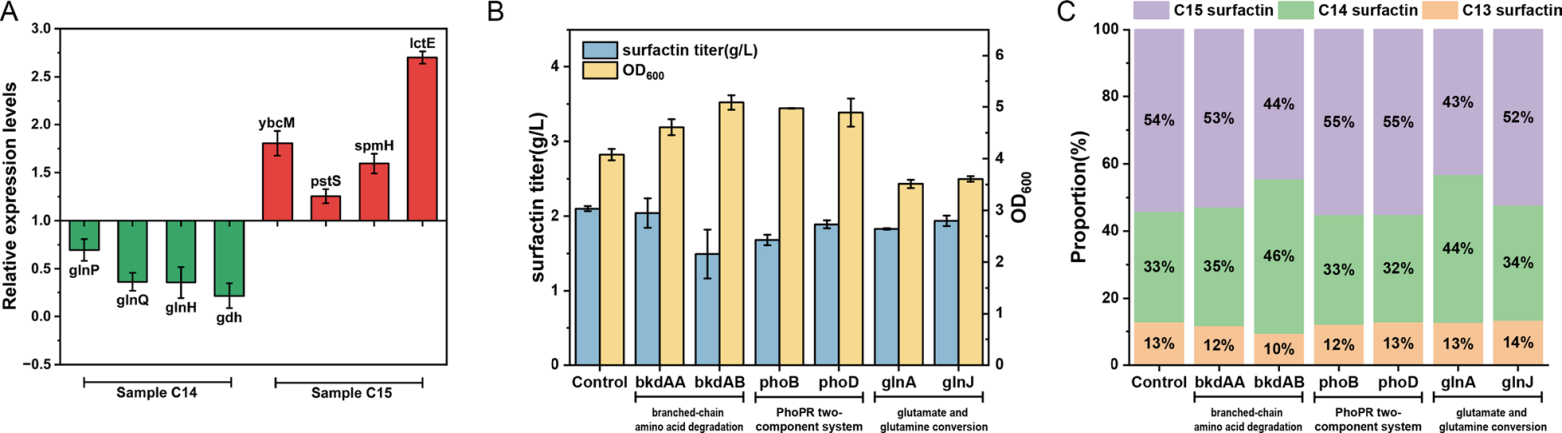
Gene transcription levels and functional gene validation. **A** RT-qPCR analysis of *glnP*, *glnQ*, *glnH* and *gdh* genes expression in sample C14 vs control, and RT-qPCR analysis of *ybcM*, *pstS*, *spmH* and *lctE* genes expression in sample C15 vs control. **B** Surfactin titer and OD_600_ of *B. subtilis Q1* by overexpressing *bkdAA*, *bkdAB*, *phoB*, *rapE*, *glnA* and *glnJ*. **C** Surfactin homologues proportion of *B. subtilis Q1* by overexpressing *bkdAA*, *bkdAB*, *phoB*, *rapE*, *glnA* and *glnJ*

To validate the transcriptome data, eight genes were randomly chosen for analysis. The transcription levels of these genes were assessed by RT-qPCR, with 16S rRNA serving as the reference gene (Fig. 5A). Two methods were employed to determine the differential transcription of these genes in *B. subtilis Q1* (Additional file 1: Table S1). The results revealed a correlation between RT-qPCR and RNA-Seq data for the selected genes, providing support for the reliability of the RNA-Seq data.

To investigate whether the regulation of surfactin homologue synthesis is influenced by the degradation of branched-chain amino acids and the transformation process of glutamate and glutamine, putative key genes were selected and overexpressed in *B. subtilis Q1*. The branched-chain α-keto acid dehydrogenase E1 subunit is encoded by both *bkdAA* and *bkdAB*, which regulate the branched-chain amino acids metabolism pathway. The overexpression of *bkdAB* resulted in a 1.3-fold increase in the proportion of C14 surfactin, whereas *bkdAA* had no significant effect on surfactin composition (Fig. 5B, C). To further confirm our postulation, the expression of the *bkdAB* gene in *B. subtilis Q1* was suppressed employing knockout technology, as demonstrated by the RNA-based strategy outlined in Additional file 1: Fig. S3. The fermentation results showed that the C15 surfactin proportion had a 15% increase, whereas the C14 surfactin components were decreased, consistent with the downregulation of *bkdAB* in sample C15 in transcriptome data. Glutamine synthetase, encoded by *glnA*, catalyzes the crucial reaction of incorporating ammonium into glutamate and is a fundamental enzyme involved in nitrogen metabolism found in all domains of life. Overexpression of gene *glnA* led to a significant increase in the percentage of C14 production, roughly 20% higher than the control group. The gene overexpression results supported our hypothesis that surfactin homologue formation is influenced by the interactions of glutamate and glutamine, in addition to branched-chain amino acid metabolism. However, the overexpression of *phoB* and *phoD* in *B. subtilis Q1* has not significantly increased the C14–C15 surfactin proportion in fermentation products. A possible explanation is that these two genes, regulated by superior regulators, do not sufficiently have major effects on surfactin synthesis. Future research could explore the impact of higher-level regulators in the *PhoPR* two-component system on surfactin synthesis.

### Structural characteristic and bioactivity determination of surfactin homologues

**Fig. 6:**
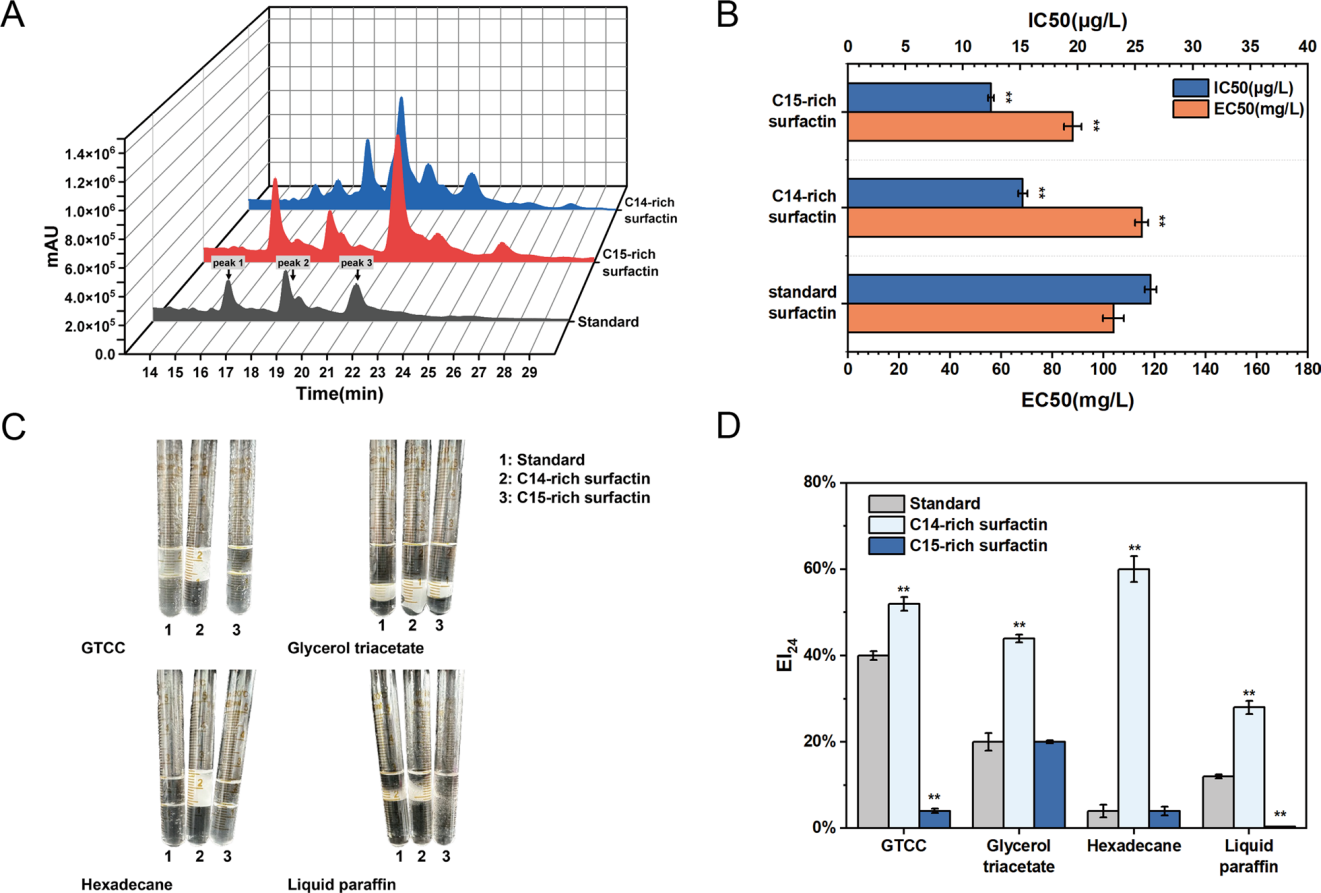
Structural characteristic and function determination of surfactin with different compositions. **A** HPLC analysis of purified C14-rich surfactin, C15-rich surfactin and commercial standard surfactin. Peaks 1, 2, and 3 represent the liquid chromatographic peaks of C13, C14, and C15 surfactin homologues, respectively. The HPLC analysis of C14–C15-rich surfactin produced in this study reveals significantly elevated peak 2–3 intensities compared to commercially available surfactin standards, respectively. **B** Hemolytic and antibacterial activity analysis of purified C14-rich surfactin, C15-rich surfactin and commercial standard surfactin. C15-rich surfactin exhibited exceptional hemolytic and antibacterial activity compared to standard surfactin. **C**,** D** Emulsifying activity of surfactin with different compositions against different substrates of GTCC, glycerol triacetate, hexadecane, and liquid paraffin. C14-rich surfactin exhibited exceptional emulsifying activity compared to standard surfactin and C15-rich surfactin, especially against hexadecane

HPLC analysis of the previous fermentation products demonstrated that the exogenous supplementation of nutrient precursor factors led to variations in peak heights at specific retention times, while no significant peaks were observed at other retention times compared to the control group (Additional file 1: Fig. S4). The finding indicates that the exogenous addition of amino acids and branched fatty acids did not induce the formation of a novel surfactin structure, but rather modulated the proportion of surfactin homologue components within the product. Previous studies have shown that different homologue compositions affect surfactin activity [28]. Branched-chain surfactin offers superior properties, including high oxidative stability, a low melting point, low viscosity, and improved flexibility, promising significant commercial potential in personal care and industrial processes [29]. To further demonstrate the effect of C14–C15 compositional occupancy on the active function, samples of C14-rich surfactin and C15-rich surfactin were prepared by exogenously adding 4 g/L valine and 2 g/L 2-methylbutyric acid, respectively, with a high purity of 75%. Component occupancy and structure were further characterized by HPLC and mass spectrometry. As shown in Fig. 6A, four components were unambiguously detected, revealing significant differences in the surfactin peaks of C14-sich surfactin, C15-rich surfactin, and standard surfactin. Notably, peak 3 was conspicuously elevated relative to the standard for C15-rich surfactin, while peak 2 was higher for C14-rich surfactin, along with an increase in the presence of additional impure peaks. To confirm structural characteristic of surfactin homologues, ESI–MS was used to determine the molecular weight information for each constituent (Additional file 1: Fig. S5). According to mass spectrometry analysis results, the major m/z of the mass [M + H] ^+^ peak corresponding to peak 2 is 1022.64, while the dominant m/z of the mass [M + H] ^+^ peak corresponding to peak 3 is 1036.66. Previous research has confirmed the presence of C13 surfactin, C14 surfactin, and C15 surfactin in peaks with molecular masses of 1008, 1022, and 1036, respectively [30]. The production strategies employed in this work were indeed effective in achieving the targeted synthesis of C14-rich surfactin and C15-rich surfactin.

To better understand the effect of fatty acid chain length on surfactin activity, the hemolytic, antibacterial, and emulsifying properties of C14-rich surfactin, C15-rich surfactin, and standard surfactin were compared. The assessment of antibacterial activity against *Micrococcus luteus*, a gram-positive bacterium commonly associated with food spoilage and conditional pathogenicity, provides insights into the potential utility of surfactin in food preservation strategies. EC50 was the concentration of surfactant required to achieve 50% hemolytic activity, while IC50 was the concentration of surfactant required to inhibit 50% indicator bacterial growth. Higher hemolytic and antibacterial activities were indicated by lower EC50–IC50 values. The EC50–IC50 values for C15-rich surfactin were 88 mg/L and 12.46 μg/mL, respectively, both lower than those for C14-rich surfactin and standard surfactin (Fig. 6B). These results suggested that higher concentration of C15 surfactin correlated with stronger hemolytic and antibacterial activity, consistent with previous research.

The amphipathic structure of surfactin confers emulsifying properties. As an emulsifier, surfactin exhibits remarkable emulsification efficiency, low irritation, and facilitates the permeation of active agents [31]. These attributes make surfactin an excellent choice for use in the cosmetics and toiletries industry. The emulsification indices (EI_24_) of the three surfactants were evaluated on different substrates (GTCC, glycerol triacetate, hexadecane, and liquid paraffin) over a 24 h period (Fig. 6C, D). In particular, C14-rich surfactin exhibited exceptional emulsifying activity on hexadecane, achieving an EI_24_ exceeding 60%, whereas C15-rich surfactin and standard surfactin displayed virtually no emulsifying capacity against hexadecane. The obtained results revealed that the C14-rich surfactin was more effective in enhancing the stability of the oil–water complex due to its superior emulsification performance.

## Discussion

In this study, the main nutritional factors affecting the synthesis of different surfactin homologues were investigated, and the further metabolic mechanism was elucidated using transcriptomics to identify the key metabolic pathways and genes involved. The fermentation results showed that supplementation of leucine significantly increased surfactin yield, and the addition of valine enhanced the C14 surfactin proportion up to above 75%. These findings were consistent with previous literature reports [32]. The exogenous addition of valine enhanced the degradation of valine and increased the proportion of even-carbon branched-chain fatty acid precursors in the fatty acid pool, leading to an increased proportion of C14 surfactin in the fermentation product. According to the study conducted by Liu et al.[9], the addition of isoleucine resulted in a C15 surfactin content of over 80% in *B. subtilis TD7*. However, in our study, the addition of isoleucine did not significantly alter the strain growth or the pH value of fermentation broth, but surfactin production cannot be identified through comparable peaks under the same HPLC conditions. This inconsistency may be due to different strains, culture conditions, and fermentation methods used in the experiment. Furthermore, this study also investigated the impact of exogenous fatty acid supplementation on the proportion of surfactin homologues. Previous research has explored the effects of adding exogenous aliphatic acids on the production of lipopeptides by *B. amyloliquefaciens Pc3 *[33]. Our study found that the regulatory effect of branched-chain fatty acids addition on the surfactin proportion was more pronounced. Specifically, the addition of even-carbon branched-chain fatty acid (isobutyric acid) increased the proportion of C14 surfactin, whereas the addition of odd-carbon branched fatty acids, such as 2-methylbutyric acid and isovaleric acid, enhanced the proportion of C15 surfactin. However, the composition of surfactin remained unaltered when supplemented with straight-chain fatty acids. This is attributed to the presence of two *FabH* isoenzymes, *FabHA* and *FabHB*, which encode 3-keto-acyl carrier protein synthase III. These isoenzymes determine the branched/straight and even/odd characteristics of the fatty acid produced, preferentially utilizing branched-chain acyl-CoA primers in *B. subtilis*. Consequently, the β-hydroxy fatty acid chain isomers involved in surfactin biosynthesis exhibit a branched structure, contributing approximately 80% to the surfactin composition. Alternatively, the specific recognition of the first C-domain in NRPS during surfactin biosynthesis may selectively catalyze the condensation of glutamate with the branched β-hydroxy fatty acid chain. The results suggest that surfactin homologue synthesis can be targeted by adjusting the amount of appropriate branched-chain fatty acids within the fatty acid pool.

Transcriptomics is useful for understanding cellular mechanisms in synthesis of different surfactin homologues. Identifying important genetic factors linked with surfactin homologue focused synthesis can be used for future strain optimization based on external regulation. Transcriptome sequencing analysis in this study demonstrated that exogenous supplementation of branched-chain amino acids and branched-chain fatty acids significantly modulates the expression of genes associated with branched-chain amino acids metabolism and the conversion of glutamate and glutamine. Branched-chain acyl-CoA, such as 2-methylbutyric acid-CoA, 2-methylpropanoyl-CoA, and 3-methylbutanoyl-CoA, are derived from branched-chain amino acids degradation. Overexpression of *bkdAB*, a gene that enhances the branched-chain amino acid degradation pathway, increased the proportion of C14 surfactin. But the overexpression of *bkdAA*, the gene encoded the same pathway enzyme with *bkdAB*, has no significant influence on surfactin component. The discrepancy can be attributed to the selectivity and specificity of enzymes encoded by different genes towards their substrates. Therefore, the upregulation of *bkdAB* expression enhances C14 surfactin production by promoting the degradation of valine to generate additional precursors for even-chain branched fatty acids.

Glutamate plays an important role in the production of surfactin, along with branched-chain amino acids. Firstly, glutamate serves as the first amino acid in the heptapeptide sequence of surfactin, whose acylation is the first step in the surfactin condensation reaction, initiating the synthesis through the condensation reaction with fatty acids. Strengthening the transport and synthesis of glutamate provides an adequate supply of precursors for surfactin. Secondly, glutamate is required in the final step of branched-chain amino acid biosynthesis, by transferring its amino group to a keto acid and forming α-ketoglutarate. This indicates a strong correlation between glutamate and branched-chain amino acids. The addition of exogenous glutamate and glutamine can increase the proportion of C14 surfactin in the fermentation product (Additional file 1: Fig. S6), aligning with previous studies exploring the impact of exogenous amino acid supplementation. However, there is currently a lack of research investigating the metabolic interplay between branched-chain amino acids and glutamate and its subsequent influence on surfactin composition. In *B. subtilis*, glutamate serves as the amino group donor, involving branched-chain amino acid synthesis. The study conducted by Thomas Rydzak et al. revealed that the absence of *glnA* gene caused a disruption in nitrogen metabolism, resulting in a significant reduction in the secretion of valine and total amino acids in *C. thermocellum* by 53%–44%, respectively [34]. The deletion of *glnA* gene led to a nitrogen starvation response, but an increase in glutamine and α-ketoglutarate levels, indicating a deregulation of nitrogen metabolism in response to *glnA* deletion. In sample C14, the upregulation of *glnA* gene expression maintained the stability of intracellular nitrogen metabolism while increasing valine secretion by high intracellular glutamate levels, thereby enhancing the production of C14 surfactin homologues. Altering the surfactin homologue composition in *B. subtilis Q1* by overexpressing the glutamine synthetase gene *glnA* further supports this view. Lipopeptides have structural diversity and broad functional capabilities, allowing them to be used in a variety of applications [35]. Cyclic lipopeptides such as surfactin, iturin, fengycin and lichenysin, which have a similar structure, have shown their best activity in industrial, environmental and medical applications [36–38]. The perspective presented in this study can not only be applied to surfactin synthesis but also serve as a guide for the regulation of other cyclic lipopeptides.

Surfactin has great potential as a replacement for traditional preservatives. Its high emulsifying properties and low irritancy make it a highly promising candidate in the field of daily chemical products. In this study, we observed a significant enhancement in the emulsifying properties of surfactin as the C14 surfactin components increased. This corresponds with the previous study, which showed that C14 surfactin displays the most favorable surface activity, leading to an optimal hydrophilic-lipophilic balance [11]. GTCC, glycerol triacetate, and liquid paraffin have been found to possess extensive applications in industries such as daily chemicals, pharmaceuticals, and food, playing crucial roles in enhancing product stability and quality. The C14-rich surfactin produced in this study displays exceptional emulsifying activity when combined with GTCC, glycerol triacetate, and liquid paraffin, surpassing the performance of commercially available surfactin products. By utilizing C14-rich surfactin, product stability can be enhanced, and the permeation of active substances can be facilitated, increasing consumer satisfaction and market competitiveness. Moreover, the widespread application of C14-rich surfactin in the emulsification field has provided related industries with innovative technological methods and solutions.

## Conclusion

In conclusion, a new perspective on the synthetic regulation mechanism of C14–C15 surfactin has been revealed. In this study, the genes *bkdAB* involved in the branched-chain amino acid pathway and *glnA* in the glutamate synthesis pathway were discovered and demonstrated to participate in the synthetic regulation of surfactin homologues. Transcriptomic analysis and overexpression experiments showed that enhancing *bkdAB* and *glnA* expression led to a 1.4-fold and 1.3-fold increase in C14 surfactin, implicating these genes as key contributors to C14 surfactin overproduction. Furthermore, the findings of this study revealed the potential of C14 surfactin in terms of emulsifying performance, offering a novel emulsifier choice for the daily chemicals, pharmaceuticals, and food industries. This study presents the first evidence of a potential connection between surfactin homologue synthesis and the conversion of glutamate and glutamine, offering valuable insights for optimizing cell factories to produce other metabolites efficiently.

### Supplementary Information

**Additional file 1**: **Table S1. **Expression of differential transcription genes in B. subtilis Q1 by RT-qPCR and RNA-Seq. **Figure S1.** Effects of carbon source and inorganic nitrogen source on surfactin production. **Figure S2. **Effects of buffer system on surfactin production. **Figure S3. **Surfactin homologues proportion of *B. subtilis Q1 *and* B. subtilis Q1-2.* **Figure S4.** The ESI–MS spectrum of C13, C14, and C15 surfactin homologue produced by *B. subtilis Q1.* **Figure S5. **Effects of exogenous addition of glutamate and glutamine on surfactin homologue proportion.

## Data Availability

All data generated or analysed during this study are included in this published article [and its supplementary information files].

## Abbreviations

IPTG: Isopropyl-β-d-thiogalactopyranoside
PBS: Phosphate buffered saline
LC–MS: Liquid chromatography mass spectrometry
HPLC: High performance liquid chromatography
