# Supplementary material for: Regulation mechanism and bioactivity characteristic of surfactin homologues with C14 and C15 fatty acid chains
Source: Microb Cell Fact. 2024 Mar 27;23:94. doi: 10.1186/s12934-024-02373-6 (PMC10967112; doi:10.1186/s12934-024-02373-6)
Supplement: Supplementary file 1 — Additional file 1: Table S1. Expression of differential transcription genes in B. subtilis Q1 by RT-qPCR and RNA-Seq. Figure S1. Effects of carbon source and inorganic nitrogen source on surfactin production. Figure S2. Effects of buffer system on surfactin production. Figure S3. Surfactin homologues proportion of B. subtilis Q1 and B. subtilis Q1-2. Figure S4. The ESI–MS spectrum of C13, C14, and C15 surfactin homologue produced by B. subtilis Q1. Figure S5. Effects of exogenous addition of glutamate and glutamine on surfactin homologue proportion. [file 12934_2024_2373_MOESM1_ESM.docx]

Regulation Mechanism and Bioactivity Characteristic of Surfactin Homologues with C14 and C15 fatty acid chains

Yumeng Su^1,a^ , Ling Gao^1,a^ , Chenyu Li, Liang Wang^a^, Huimin Zhou^a^, Chenhao Zhang^a^, Xiaole Xia*

^a^ Key Laboratory of Industrial Biotechnology, Ministry of Education, School of Biotechnology, Jiangnan University, Wuxi, Jiangsu214122, People’s Republic of China.

^1^Author Contributions: Yumeng Su and Ling Gao contributed equally to this work.

*Correspondence

Xiaole Xia, the Key Laboratory of Industrial Biotechnology, Ministry of Education, School of Biotechnology, Jiangnan University, 1800 Lihu Road, WuXi 214122, PR China

Tel./Fax: +86-0510-85327270

E-mail: [xiaolexia@jiangnan.edu.cn](mailto:xiaolexia@jiangnan.edu.cn)

**Table S1. Expression of differential transcription genes in *B. subtilis Q1* by RT-qPCR and RNA-Seq.**

| Sample | Gene name | 2 ^-(∆∆Ct)^ (RT-qPCR) | Log_2_FoldChange (RNA-Seq) |
| --- | --- | --- | --- |
| C14 vs Control | *glnP* | 0.70 | -7.76 |
|  | *glnQ* | 0.36 | -5.78 |
|  | *glnH* | 0.36 | -5.06 |
|  | *gdh* | 0.22 | -5.68 |
| C15 vs Control | *ybcM* | 1.81 | 7.65 |
|  | *pstS* | 1.25 | 5.46 |
|  | *spmH* | 1.59 | 4.41 |
|  | *lctE* | 2.70 | 5.09 |

Note: In the control group, 2 ^-(∆∆Ct)^ equals 1, and Log_2_FoldChange equals 0.

**Figure S1. Effects of carbon source and inorganic nitrogen source on surfactin production.** A: Surfactin titer and OD_600_ of *B. subtilis Q1* at different carbon sources; B: Surfactin homologues proportion of *B. subtilis Q1* at different carbon sources; C: Surfactin titer and OD_600_ of *B. subtilis Q1* at different inorganic nitrogen sources; D: Surfactin homologues proportion of *B. subtilis Q1* at different inorganic nitrogen sources.


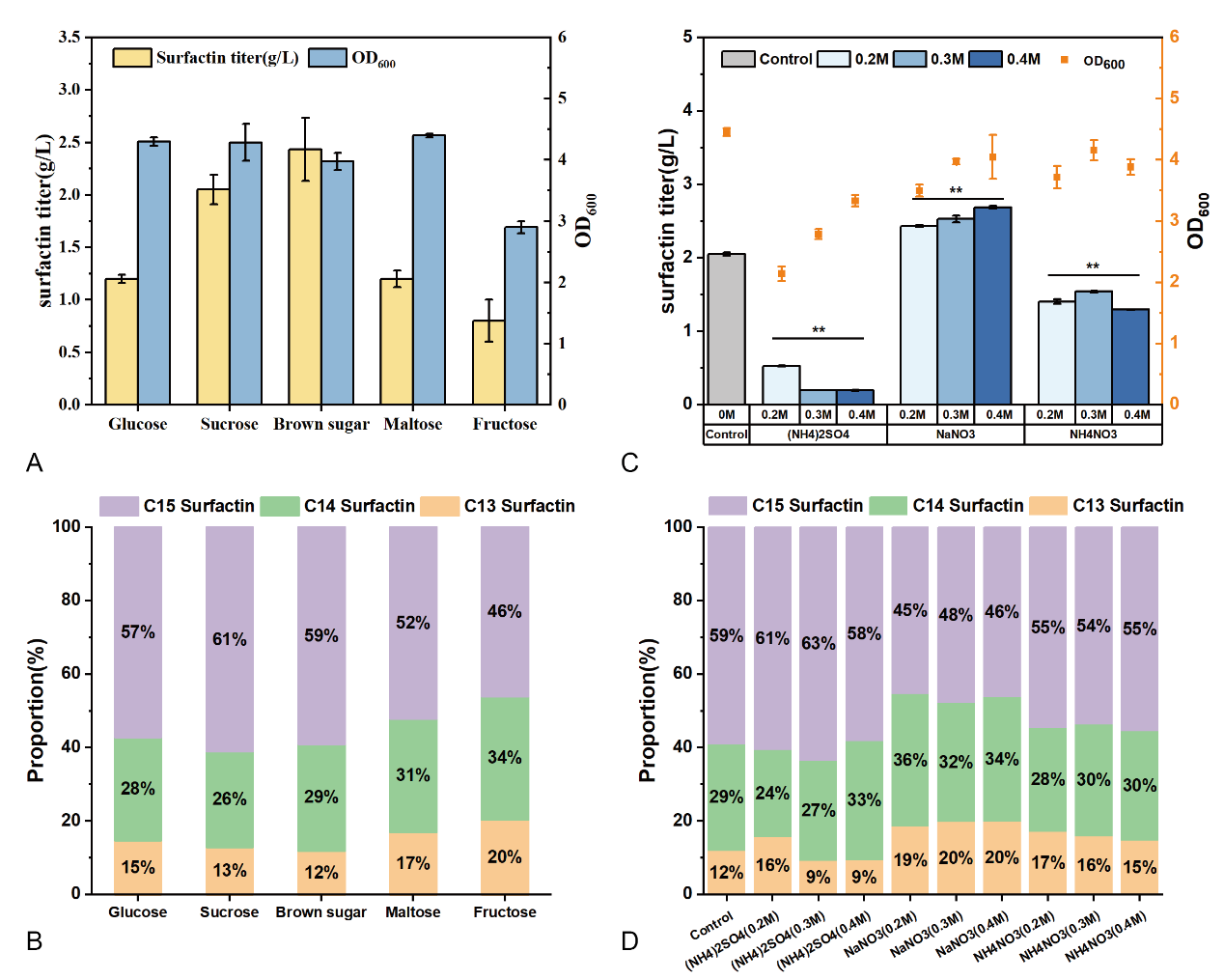


**Figure S2. Effects of buffer system on surfactin production.**

**

**

**Figure S3. Surfactin homologues proportion of *B. subtilis Q1* and *B. subtilis Q1-2.***





**Figure S4. HPLC analysis of the fermentation products with the exogenous supplementation of nutrient precursor factors.** A: HPLC analysis of surfactin products with the exogenous supplementation of amino acids. B. HPLC analysis of surfactin products with the exogenous supplementation of branched-chain fatty acids.


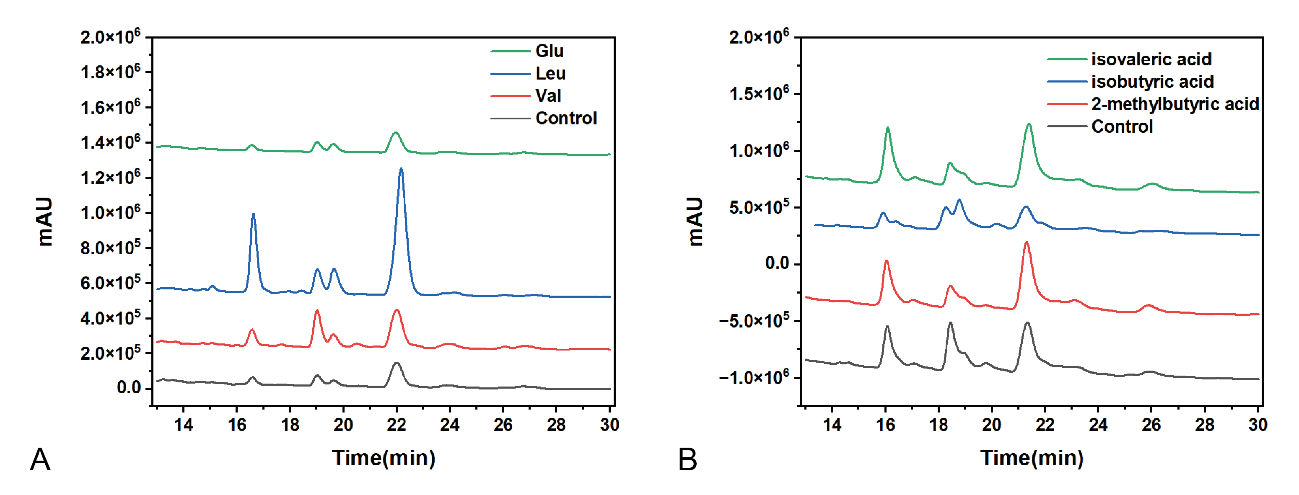


**Figure S5. The ESI–MS spectrum of C13, C14, and C15 surfactin homologue produced by *B. subtilis Q1*.** A: The ESI–MS spectrum of C13 surfactin; B: The ESI–MS spectrum of C14 surfactin; C: The ESI–MS spectrum of C15 surfactin.


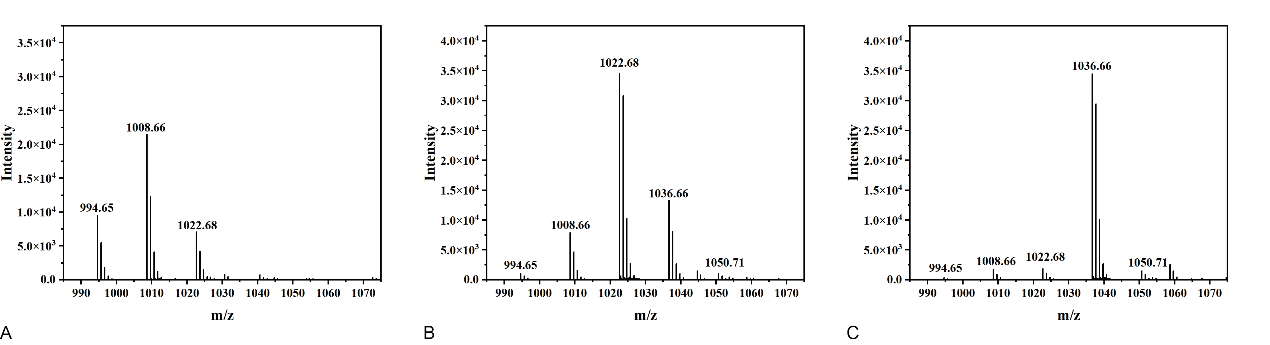


**Figure S6. Effects of exogenous addition of glutamate and glutamine on surfactin homologue proportion.**
